# A naturalistic study of high-dose unilateral ECT among severely depressed inpatients: how does it work in the clinical practice?

**DOI:** 10.1186/s12888-016-1095-z

**Published:** 2016-11-11

**Authors:** Lucas P. C. Alves, Thiago F. V. Freire, Marcelo P. A. Fleck, Neusa S. Rocha

**Affiliations:** 1 Universidade Federal do Rio Grande do Sul, Hospital de Clínicas de Porto Alegre, Programa de Pós Graduação em Psiquiatria e Ciências do Comportamento – RS – Brazil, 2350, Ramiro Barcelos Street, Porto Alegre, Rio Grande do Sul Brazil; 2 Universidade Federal do Rio Grande do Sul, Programa de Pós Graduação em Psiquiatria e Ciências do Comportamento, Hospital de Clínicas de Porto Alegre-RS-Brazil, 2350, Ramiro Barcelos Street, Porto Alegre, Rio Grande do Sul Brazil; 3 Universidade Federal do Rio Grande do Sul, Hospital de Clínicas de Porto Alegre, Programa de Pós Graduação em Psiquiatria e Ciências do Comportamento – RS –Brazil, 2350, Ramiro Barcelos Street, Porto Alegre, Rio Grande do Sul Brazil

**Keywords:** Electroconvulsive therapy, Depressive disorder, Naturalistic Study, Pragmatic Clinical Trials as Topic

## Abstract

**Background:**

Naturalistic studies can be useful tools to understand how an intervention works in the real clinical practice. This study aims to investigate the outcomes in a naturalistically treated depressed inpatients cohort, who were referred, or not, to unilateral ECT.

**Methods:**

Depressed adults according to MINI admitted in a psychiatric unit were divided in unilateral ECT treated and non-ECT treated. Main outcomes were: depression improvement in Hamilton Rating Scale for Depression (HDRS-17) scores; response (HDRS-17 improvement ≥50 %); remission (HDRS-17 score ≤7); length of hospitalization.

**Results:**

Forty-three patients were included in unilateral ECT group and 104 in non-ECT group. No differences of psychotic symptoms, melancholic features or past maniac episode were found between groups. Unilateral ECT group had a mean HDRS-17 score higher than non-ECT group at admission (ECT: 25.05 ± 1.03; non-ECT: 21.61 ± 0.69; *p =* 0.001), but no significant difference was found at discharge (ECT: 7.70 ± 0.81; non-ECT: 7.40 ± 0.51; *p =* 0.75). Unilateral ECT group had a larger HDRS-17 score reduction during treatment (ECT: 18.24 ± 1.18; non-ECT:14.20 ± 0.76; *p =* 0.004). There were no significant differences in response and remission rates between groups. Unilateral ECT group had longer mean duration of hospitalization in days (ECT: 35.48 ± 2.48; non-ECT: 24.57 ± 1.50; *p <* 0.001), but there were no difference in mean time of treatment (ECT group:27.66 ± 1.95; non-ECT: 24.57 ± 1.50; *p =* 0.25).

**Conclusions:**

Unilateral high-dose ECT is still a useful treatment option, in the real world clinical practice, to reduce the intensity of depressive symptoms in highly depressed inpatients.

## Background

Electroconvulsive therapy (ECT) is a well-documented method for the treatment of several psychiatric conditions [1] and several meta-analyses have proven its efficacy and safety in the treatment of depressive disorders [2–4]. However, extrapolating the findings of randomized clinical trials (RCTs) and meta-analyses to clinical practice is still a challenge for clinicians, especially when analyzing patients with high incidence of somatic and psychiatric comorbidities.

Therefore, when an experienced clinician indicates ECT for a patient, he or she may do so based on evidence regarding a particular group of patients. In this context, many indications can be listed. For instance, patients with the psychotic subtype of depression have higher response rates to ECT than do patients without psychosis [5] and severely ill patients diagnosed with melancholic depression also have excellent response rates to ECT [6]. Response rates may also be higher among the elderly [7]. Furthermore, the fast clinical response produced, which is often faster than the medication-induced response [8], makes ECT a first-line treatment in urgent clinical situations such as severe suicidality, severe psychosis, catatonia, and malnutrition in patients with food refusal secondary to depressive illness [8, 9].

Once the patients have clinical indications to ECT, electrode position is also another issue to be considered. Bitemporal ECT is the most commonly used electrode placement in the world [10]. However it causes more cognitive deficits when compared to unilateral ECT [11]. Based on dosage, unilateral ECT is less effective than bilateral ECT, but several trials demonstrated that, when delivered in high doses (e.g. 6× of seizure threshold), unilateral ECT can be as effective as bitemporal ECT, with fewer cognitive adverse effects [12–15]. Recently, non-inferiority trial also showed that even twice-week unilateral ECT was not-inferior to bitemporal ECT in depressed patients [16].

Hence, there are many variables that one must consider when deciding to indicate ECT for a depressed patient. However, due to ethical reasons such as patient consent and clinical decisions (e.g., whether to perform invasive therapies on less severely afflicted patients), ECT is still mainly used to treat severely depressive patients or patients resistant to conventional therapy. Patients who receive ECT typically have mean scores greater than 30 on the Hamilton Depression Rating Scale - 17 items (HDRS-17) [17].

Although naturalistic studies were not designed to prove the efficacy or efficiency of a treatment, they can be a useful tool to understand how an intervention works in real clinical practice. For example, a naturalistic multi-center study conducted in 12 psychiatric hospitals in Germany showed that the results found in phase III trials may be different from those found in patients in “the real world”, probably because of the strict exclusion criteria of randomized controlled trials (RCTs) [18]. Moreover, in the field of ECT, response rates in community hospitals may be less robust than those found in clinical trials [18–20].

Although some naturalistic studies regarding ECT have already been published [18, 21–24], there are still some limitations that we must consider when transposing these results to clinical practice. First, the improvement of both ECT techniques and antidepressant therapy has resulted in a difficult interpretation of older studies [21]. For example, though the UK ECT review group have reported that bilateral electrode placement is more effective than right unilateral placement, newer studies have shown that there are no differences between high-dose unilateral ECT and bilateral ECT [10, 12, 13, 15]. On the other hand, naturalistic studies conducted more recently either had ECT as a secondary outcome [18, 23, 24], or did not have a control group [22]. Furthermore, as far as we know, all of the studies were conducted in developed countries, and extrapolation of these results to undeveloped or developing countries is sometimes controversial.

In this context, studies that evaluate current protocols by comparing severely ill patients that need ECT in real clinical practice with those that do not may be useful for clinicians who face the challenge of indicating ECT. Therefore, the objective of this study is to evaluate the outcomes (depression severity, response, remission, time of hospitalization) of severely depressed inpatients who were referred to receive high-dose unilateral ECT when compared to patients who were not referred to such treatment.

## Methods

We conducted a naturalistic prospective cohort study comparing depressive inpatients who underwent two different treatment strategies, decided by the assistant psychiatrist, not involved in the research group: ECT (either alone or with antidepressant pharmacotherapy) and antidepressant pharmacotherapy alone. The study was conducted at the psychiatric unit of the Hospital de Clínicas de Porto Alegre, Porto Alegre, Southern Brazil, between May 2011 and April 2013. Hospital de Clínicas is a university general hospital of tertiary care. Its psychiatry unit is inserted in the hospital, attending patients arising from both public health system and health insurance plans. In addition, the hospital has a strong tradition of performing ECT, being the only public hospital of its state that performs ECT, a unit of reference in the south of Brazil. Informed consent was obtained from all patients and the Ethical and Scientific Committee approved the project (n° 10-265). This study is part of a broader project that has the aim of following severe mental ill patients after a psychiatric hospitalization in a tertiary university hospital.

Our main outcome, depression improvement, was assessed through a) the difference between the Hamilton Depression Rating Scale-17 items (HDRS-17) [25] scores at admission and discharge, and b) the percentage of patients with responses (≥50 % improvement in the HDRS-17 total score) and remissions (HDRS-17 score ≤7) at discharge.

Secondary outcomes included the duration of hospitalization, as measured by the number of days between admission and discharge. A corrected time of hospitalization for the ECT group was also calculated using the difference in the number of days between discharge and the day of the first ECT session. No corrections were made in the non-ECT group because, since we evaluated patients in a tertiary university hospital, pharmacological therapy starts since the first day of hospitalization. Furthermore, we evaluated Clinical Global Impression (CGI), Global Assessment of Functioning Scale (GAF), and Brief Psychiatric Rating Scale (BPRS), both in admission and discharge. The difference between its scores at admission and discharge (delta) were also compared for both groups.

The psychiatric diagnosis of each patient was made through a structured interview. The instrument used was the Mini-International Neuropsychiatric Interview (MINI) [26], which is based on the DSM-IV criteria. The MINI was applied by psychiatrists who were not involved in patient care. Diagnoses were made one day after admission. The same physicians who detected major depression using the MINI also determined the HDRS-17 score at admission. Psychiatric evaluations were performed again by another physician one day before discharge.

Patients were included based on the following criteria: a) 18 years of age or more, b) diagnosed with major depression according to the MINI, and c) stayed in the hospital for at least 7 days. Patients who had drug or alcohol addictions or dependence as the main diagnosis at admission or whose diagnosis and severity scales could not be obtained at both admission and discharge were excluded from the study. Patients’ demographic characteristics were obtained through a questionnaire and from medical records. Presence of past maniac episode, melancholic depression and psychotic symptoms were also evaluated by MINI.

Clinical indications of ECT were based on Task Force Report of the American Psychiatric Association [27], such as resistance of the symptoms to standard therapy, presence of catatonic symptoms, long episode duration, psychotic depression, or acute suicide risk. Patients were referred for ECT by clinical indication of the assistant psychiatrist, without interference from the researchers. We evaluated patients’ records described by assistant psychiatrist to determine their clinical indications. Main interventions are described below.

ECT was performed only after informed consent was obtained. All patients received high-dose (6 times the convulsive threshold), brief-pulse, right unilateral ECT using the d’Elia position [8]. ECT was performed using Spectrum 5,000Q (MECTA) [28]. Patients were anesthetized with thiopental (3 mg/kg intravenously) along with succinylcholine (1 mg/kg intravenously) as a muscle relaxer. The stimulus intensity was determined by the titrated strategy. The ictal response was recorded with an electroencephalogram, and the cuff method was used to monitor motor convulsive activity. An adequate seizure was defined as a myoclonus of 20 s or longer or an electroencephalographic seizure of 25 s or longer.

ECT procedures were established according to the protocol of Hospital de Clínicas de Porto Alegre, and ECT was performed as follows: three times a week, in the morning, in an ambulatory surgical center, in the presence of a psychiatrist and an anesthesiologist, and with electrocardiographic and electroencephalographic monitoring [29].

The number of ECT sessions was based on the clinical judgment of the assistant psychiatrist for each patient, with a median between 8 and 12 sessions.

Psychopharmacological management was also based on the clinical decision of the assistant psychiatrist and was based on the general principles of dose optimization, the combination of two antidepressants, potentialization with different strategies (e.g., lithium, atypical antipsychotics), or changing to another antidepressant [30].

For the analysis, patients were divided into two groups: those who received ECT (ECT group) and those who did not receive ECT (non-ECT group).

### Statistical analysis

We calculated a power of 80 % for our sample to identify a difference between groups of 4 points on the HDRS-17. The mean difference with 95 % confidence intervals (CIs) for each exposure of the baseline was estimated using the Statistical Package for the Social Sciences software (SPSS, from IBM Company©, United States, version 19). The Shapiro-Wilk test of normality was used to test the normality of the continuous variables between the groups. Since all variables were considered to have a normal distribution, we used parametric tests. The difference of improvement between groups was calculated by *t*-tests for independent samples. Potential baseline confounders were controlled using Pearson’s correlations and *t*-tests for independent samples, with the HDRS-17 total score improvement as the dependent variable. Response and remission rates were compared by chi-squared tests.

## Results

**Fig. 1:**
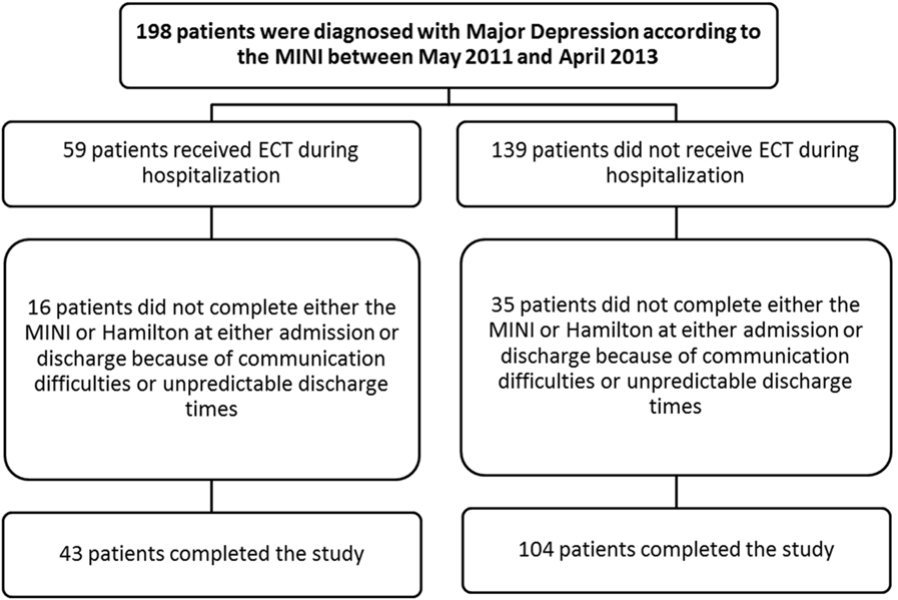
Number of inpatients included in the cohort and analysis. MINI: Mini-International Neuropsychiatric Interview; ECT: Electroconvulsive therapy

A total of 147 patients were included in the study, with 43 in the ECT group and 104 in the non-ECT group (Fig. 1). The baseline characteristics of both groups are presented in Table 1. Comparing to non-ECT group, ECT group was older, with greater percentage of white females, and a higher percentage of previous ECT. We found no difference of presence of psychotic symptoms, maniac episode in the past and melancholic features between groups.

**Table 1:** Characteristics of the 147 psychiatric inpatients in the study

Characteristics	General (*n =* 147)	ECT group (*n =* 43)	Non-ECT group (*n =* 104)	*P* (between groups)
Age (±SD)	45.41 (±14.59)	51.12 (±14.85)	43.07 (±13.88)	0.002*
Number of previous hospitalizations (±SD)	3.33 (±4.88)	2.9 (±3.13)	5.51 (±5.45)	0.51
Age at the last hospitalization (±SD)	41.99 (±13.90)	48.64 (±15.18)	39.39 (±1.6)	0.004*
Sex				
Male (%)	57 (38.8)	11 (25.6)	46 (44.2)	0.03*
Ethnicity				
White (%)	125 (85)	41 (95.3)	84 (80.8)	0.02*
Other (%)	22 (15)	2 (4.7)	20 (19.2)	
Had ECT before				
Yes (%)	25 (17)	14 (32.5)	11 (10.5)	0.001*
Presence of Melancholy				
Yes (%)	99 (72.8)	33 (80.5)	66 (69.5)	0.132
Presence of Psychotic Symptoms				
Yes (%)	47 (33.1)	17 (41.5)	30 (29.7)	0.177
Maniac Episode in the Past				
Yes (%)	50 (35.5)	17 (42.5)	33 (32.7)	0.272
Number of medications in use:				
Anti-depressives				
0 (%)	53 (35.8)	37 (40.2)	16 (44.4)	0.9
1 (%)	62 (41.9)	45 (48.9)	17 (48.4)	
2 (%)	12 (8.1)	9 (9.8)	3 (8.3)	
3 (%)	1 (0.7)	1 (1.1)	0	
Anticonvulsants				
0 (%)	105 (70.9)	33 (84.6)	72 (75.8)	0.46
1 (%)	26 (17.6)	5 (12.8)	21 (22.1)	
2 (%)	3 (2)	1 (2.6)	2 (2.1)	
Lithium				
Yes (%)	25 (16.9)	5 (12.8)	20 (21.3)	0.19
Typical anti-psychotics				
0 (%)	90 (60.8)	60 (65.9)	30 (76.9)	0.27
1 (%)	32 (21.6)	26 (28.6)	6 (15.4)	
2 (%)	8 (5.4)	5 (5.5)	3 (7.7)	
Atypical anti-psychotics				
0 (%)	49 (33.1)	38 (41.3)	11 (28.2)	0.13
1 (%)	75 (50.7)	51 (55.4)	24 (61.5)	
2 (%)	7 (4.7)	3 (3.3)	4 (10.3)	
Benzodiazepines				
0 (%)	82 (55.4)	54 (58.1)	28 (71.8)	0.29
1 (%)	49 (33.1)	38 (40.9)	11 (28.2)	
2 (%)	1 (0.7)	1 (1.1)	0	

*ECT* Electroconvulsive therapy, *SD* Standard deviation

* *P* < 0.05

According to patients’ records described by the assistant psychiatrist, 42 patients received ECT due to refractoriness of symptoms; only one patient received ECT because of intolerable side effects of pharmacotherapy. Among the 14 patients in the ECT group who had received ECT previously, 7 of them were admitted with the specific intention of receiving ECT again.

### Improvement of scores of the depression scales between the ECT and Not-ECT Groups

**Fig. 2:**
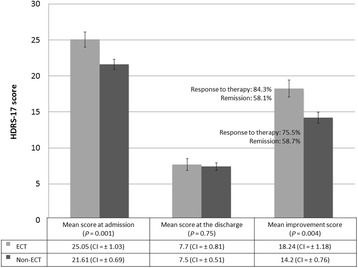
Mean Hamilton Depression Rating Scale-17 items (HDRS-17) score for each group at admission, discharge, and difference between admission and discharge. ECT: Electroconvulsive therapy; CI: Confidence interval

Figure 2 shows the HDRS-17 scores of each group at admission and discharge. The mean HDRS-17 score of the ECT group (25.05, CI: ±1.03) was significantly different from the score of the non-ECT group (21.61, CI: ±0.69) at admission (*P* = 0.001). However, the scores were not significantly different at discharge (ECT group: 7.70, CI: ±0.81; non-ECT group: 7.50, CI: ±0.51; *P* = 0.75). The improvement from admission to discharge was significant in both groups (*P* < 0.001 for both groups).

When comparing the HDRS-17 improvement scores between admission and discharge, the mean reduction of the HDRS-17 total score was 18.24 points (CI: ±1.18) in the ECT group compared to 14.20 points (CI: ±0.76) in the non-ECT group. This difference was statistically significant (*P =* 0.004).

Response rates were 84.3 % in the ECT group and 75.5 % in the non-ECT group (*P =* 0.125), and remission was 58.1 % in the ECT group and 58.7 % in the non-ECT group (*P =* 0.55), when analyzing by HDRS-17.

### Duration of hospitalization

**Fig. 3:**
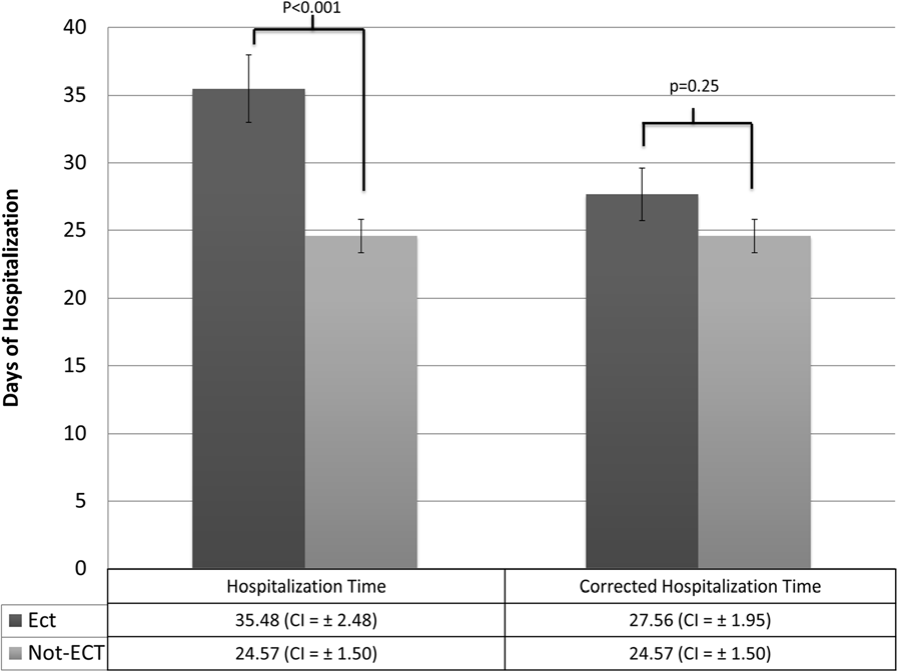
Mean duration of hospitalization of subjects in the ECT and non-ECT groups. ECT: Electroconvulsive therapy; CI: Confidence interval

The mean duration of hospitalization was 35.5 (CI: ±2.5) days for the ECT group and 24.6 (CI ±1.50) days for the non-ECT group (*P* < 0.001). When we corrected the ECT group’s duration of hospitalization by using the difference between the time of the first ECT session and that of hospital discharge, the mean time was 27.6 days (CI: ±1.95), which was not statistically different from that of the non-ECT group (*P =* 0.25). These data are summarized in Fig. 3.

### Control of potential confounders and evaluation of secondary outcomes

Potential confounders (age, age of the last hospitalization, number of previous hospitalizations, sex, ethnicity, and previous ECT) are shown in Table 2. These values were calculated using the mean difference of the HDRS-17 total score improvement between groups as the dependent variable. None of the potential confounders was statistically significant in our study. Although there was a significant difference in the CGI, GAF and BPRS scales between groups in the admission, the delta scores between discharge and admission were not significant between groups. These data are presented in Table 3.

**Table 2:**
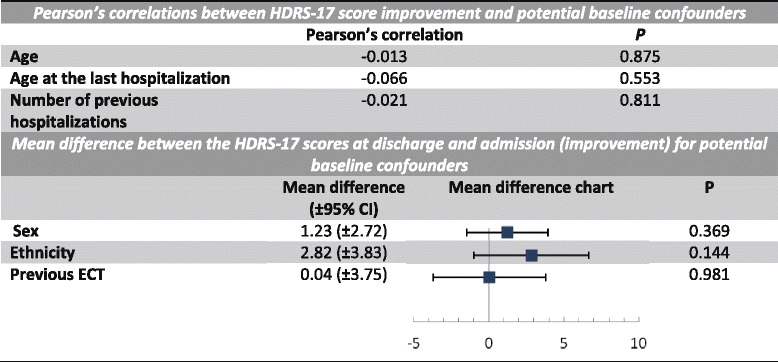
Control of potential confounders

*HDRS-17* Hamilton Depression Rating Scale-17 items, *CI* Confidence interval, *ECT* Electroconvulsive therapy

**Table 3:** Secondary outcomes evaluation between ECT and Not-ECT group

	Admission			Discharge			Delta		
	ECT	Not-ECT	*P*	ECT	Not-ECT	*P*	ECT	Not-ECT	*P*
Mean CGI (±CI)	5.71 (±0.09)	5.12 (±0.11)	0.002*	3.43 (±0.21)	3.14 (±0.12)	0.21	2.25 (±0.18)	1.92 (±0.15)	0.14
Mean GAF (±CI)	30.07 (±2.06)	35.52 (±1.62)	0.06	60.16 (±2.97)	64.97 (±1.64)	0.13	-29.64 (±4.07)	-29.45 (±2.41)	0.22
Mean BPRS (±CI)	27.37 (±1.43)	23.38 (±0.1)	0.03*	10.02 (±1.2)	8.71 (±0.55)	0.26	17.5 (±1.67)	14.72 (±0.1)	0.97

*CGI* Clinical Global Impression, *GAF* Global Assessment of Functioning, *BPRS* Brief Psychiatric Rating Scale

* *P* < 0.05

## Discussion

The main finding of the present study was that, by a naturalistic design, high-dose unilateral ECT, in clinical practice, is still a useful method to reduce the intensity of depressive symptoms in highly depressed inpatients. We were able to show that the patients who received ECT were even more depressed at admission, and yet they showed very similar outcomes at discharge (HDRS-17, response and remission) compared to patients who did not receive ECT. This happened due to a longer mean duration of hospitalization and a greater reduction of depressive symptoms, also because the ability to reduce the score in ECT patients was the greatest (since they were more depressed in the baseline). Although naturalistic studies have some limitations such as lack of randomization, it has the advantage of better representing the “real world” clinical practice [31]. In contrast, randomized clinical trials, which are the gold standard in the evaluation of efficacy, have often a limited capacity of generalization [18]. By this point of view, we could say that the results of these two models complete each other. Nevertheless, we could say that our results are consistent with the results found in similar studies [17, 21], though our study has the added advantage of having a control group that, in the same setting, did not receive ECT. A similar result was also found in recent clinical trial, although it only included depressed bipolar patients [32].

Furthermore, the presence of melancholia, bipolar disorder or psychotic depression was no different between groups, which shows that apparently this is not the most relevant data for a clinician to refer a patient to ECT. Clinicians are probably more likely to opt for ECT based on a broader clinical presentation of the patient, including depression severity, clinical global impression and a history of previous ECT sessions. This is consistent with recent meta-analysis that showed that patient characteristics such as age, psychosis, and melancholic features are less likely to be good predictors of ECT response [33]. However, we do not have information regarding past medical history of the patients, which may also have influenced clinicians to opt for ECT.

The gap between admission and first ECT session also shows that refractoriness to antidepressant therapy could also play an important role in the choice of treatment, even for severely affected patients. Since the decision to refer patients to ECT was made by the assistant psychiatrist, other factors than refractoriness could also cause the gap between admission and ECT; however, we have insufficient data to answer this question.

Our study has some limitations. First, our sample size does not have enough power for subgroup analysis to be performed, though we were able to find results with statistical significance for the primer outcome. Second, our study was a naturalistic one, rather than a randomized clinical trial. This design may create a conservative bias (“against ECT”) since the more severe patients were not randomly allocated, but instead tended to predominate in the ECT group. However, this design is more susceptible to confounding bias due to comorbidities between the groups, such as personality disorders comorbidities or other psychiatric comorbidities. Third, a wide variety of pharmacological strategies was used in both groups, and was not controlled for between groups. For example, although we were able to make a corrected length of hospitalization for the ECT group, we could not do the same for the non-ECT group (i.e., to compute the length of hospitalization of the effective pharmacologic treatment). Fourth, since we made the diagnostic evaluation only in the hospital admission, we were not able to evaluate diagnostic changes during the hospitalization, such as switches to mania after either ECT or pharmacologic treatment. Fifth, since ECT was performed in a general hospital that is unit of reference for other psychiatry centers, the conclusions of this study may be not extendable to settings of attention. At the same time, our study presents data from a clinical setting based on decisions made in the real world.

## Conclusions

Even though the depressed patients who underwent ECT were more severe at hospital admission, we found that at discharge they were similar to less severe patients (measured at baseline) who did not need to receive ECT. Clinical indications for ECT appeared to be based on the depression severity, clinical global impression and a history of previous ECT sessions. These findings support ECT as a good choice of treatment for highly depressed patients in the real world.

## Abreviations

BPRS: Brief psychiatric rating scale
CGI: Clinical global impression
CI: Confidence interval
ECT: Electroconvulsive therapy
GAF: Global assessment of functioning scale
HDRS-17: Hamilton depression rating scale - 17 items
MINI: Mini-international neuropsychiatric Interview
RCTs: Randomized clinical trials
